# Laparoscopic cholecystectomy plus endoscopic retrograde direct cholangioscopy: an integrated strategy for Mirizzi syndrome

**DOI:** 10.1055/a-2578-2273

**Published:** 2025-05-06

**Authors:** Zhu-Hui Liu, Yu-Tong Yao, Guang-Ming Xiang, Wei-Hui Liu

**Affiliations:** 1 89669 Department of Gastroenterology and Hepatology, Sichuan Provincial People’s Hospital, School of Medicine, University of Electronic Science and Technology of China, Chengdu, China; 2 89669 Department of Geriatric Comprehensive Surgery, Sichuan Provincial People’s Hospital, School of Medicine, University of Electronic Science and Technology of China, Chengdu, China

Mirizzi syndrome, a rare but serious complication of cholelithiasis, is difficult to differentiate from cholangitis and choledocholithiasis. Despite the help of preoperative imaging, more than 50% of cases of Mirizzi syndromes are still diagnosed intraoperatively 1 . Even if the preoperative diagnosis is clear, intraoperative cholangiography, choledochoscopy, or intraductal ultrasonography may still be needed to confirm the diagnosis and determine the presence, size, and location of the fistula 2 . Therefore, we propose an integrated strategy for suspected Mirizzi syndrome, this being to perform endoscopic retrograde direct cholangioscopy (ERDC) 3 4 simultaneously during laparoscopic cholecystectomy, to simplify the diagnosis and treatment process while ensuring safety ( Video 1 ).

Laparoscopic cholecystectomy plus endoscopic retrograde direct cholangioscopy are performed, providing an integrated strategy for Mirizzi syndrome.Video 1

A 41-year-old man in our hospital was suspected of having Mirizzi syndrome. Laparoscopic cholecystectomy and ERDC were performed simultaneously without radiography support. First, direct vision through the cholangioscope revealed the cystic duct stone and the compressed common hepatic duct, which confirmed the diagnosis of Mirizzi syndrome ( Fig. 1 ). The stone was removed using a slim extraction basket and saline irrigation after electrohydraulic lithotripsy ( Fig. 2 ). Under cholangioscopic visualization, we confirmed the common bile duct (CBD) was a sealed cavity without residual stones, which also meant this patient had type I Mirizzi syndrome ( Fig. 3 ). A porcelain gallbladder could be observed through the unobstructed cystic duct, while laparoscopically the gallbladder appeared luminous ( Fig. 4 ). Guided by the light of the cholangioscope, we were able to accurately identify the cystic duct, CBD, and common hepatic duct ( Fig. 5 ). After the gallbladder triangle had been dissected, the gallbladder was successfully excised. The patient made a full recovery, with no complications reported.

**Fig. 1:**
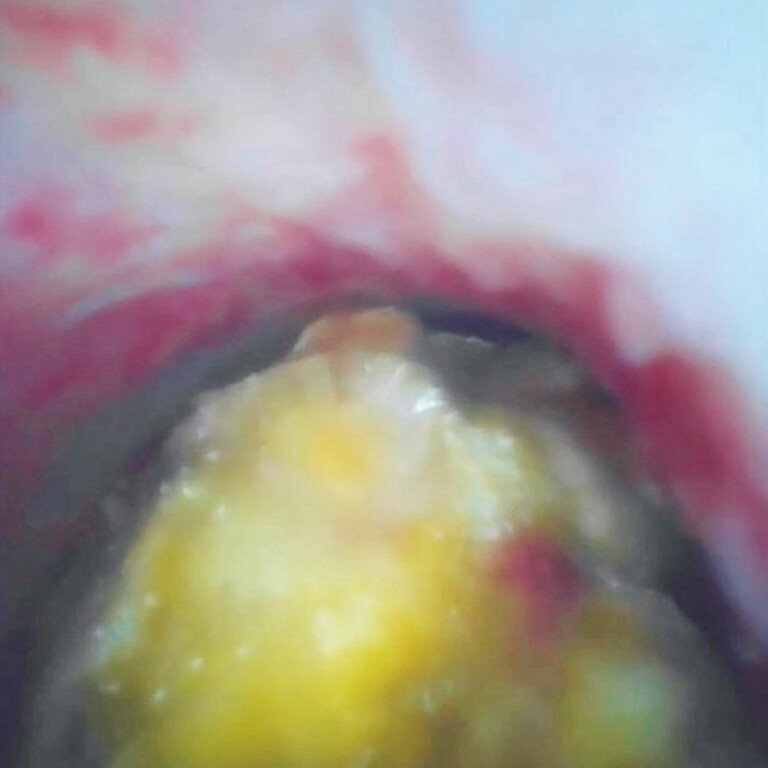
Cholangioscopic view showing the obstructing cystic duct stone.

**Fig. 2:**
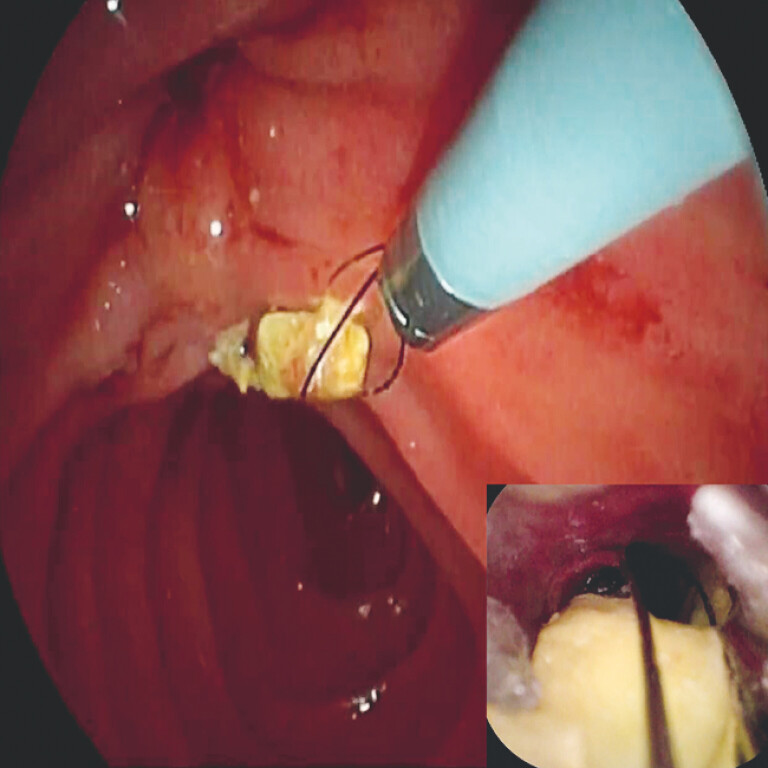
Endoscopic view showing the cystic duct stone being removed with a slim extraction basket and saline irrigation after electrohydraulic lithotripsy had been performed.

**Fig. 3:**
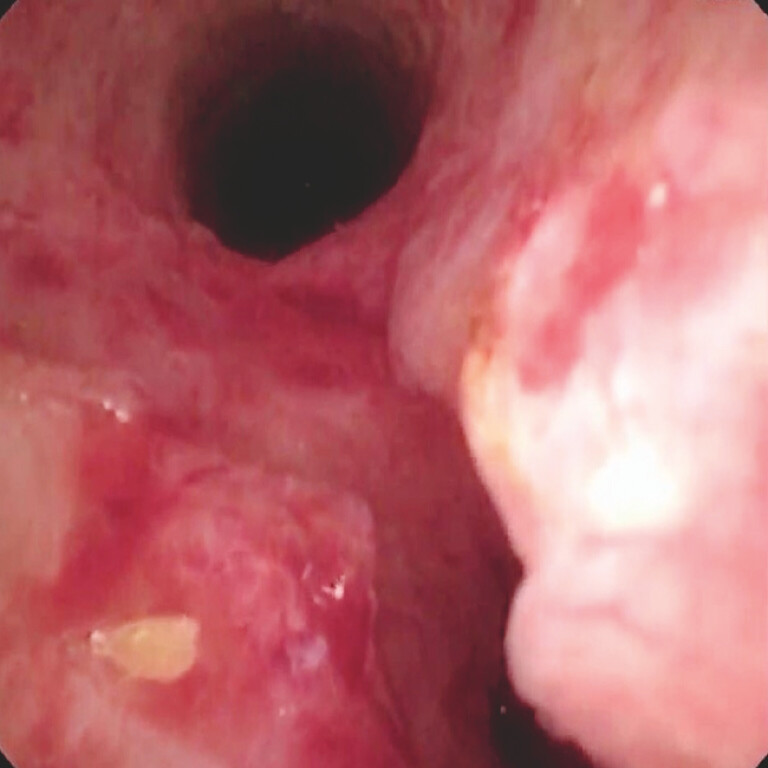
Cholangioscopic view showing no evidence of leaks or residual stones in the common bile duct.

**Fig. 4:**
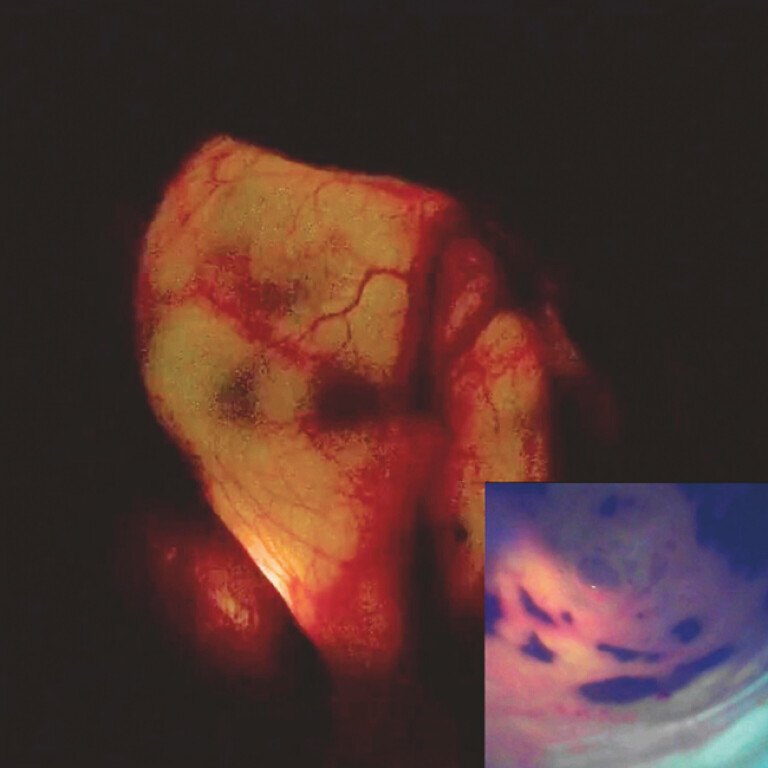
Laparoscopic view showing luminosity of the gallbladder (inset: cholangioscopic image of the porcelain gallbladder).

**Fig. 5:**
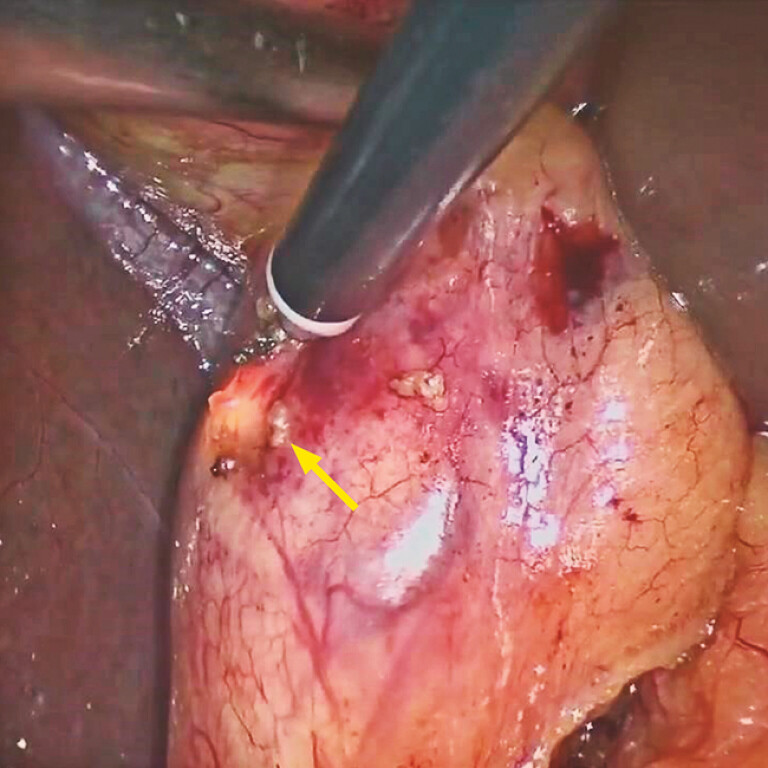
Image during laparoscopic cholecystectomy showing how the cholangioscope provided a direct indication of the location of the bile duct.

In our practice, ERDC clarified the diagnosis and classification of Mirizzi syndrome, removed the obstructing stones, provided direct guidance for laparoscopic gallbladder triangle dissection, and avoided the huge trauma caused by open surgery. Our strategy may be a novel, safe, efficient, and economical solution to Mirizzi syndrome, achieving both diagnostic and therapeutic goals.

Endoscopy_UCTN_Code_TTT_1AR_2AH

## Notes

Endoscopy E-Videos https://eref.thieme.de/e-videos
*E-Videos* is an open access online section of the journal *Endoscopy* , reporting on interesting cases and new techniques in gastroenterological endoscopy. All papers include a high-quality video and are published with a Creative Commons CC-BY license. Endoscopy E-Videos qualify for HINARI discounts and waivers and eligibility is automatically checked during the submission process. We grant 100% waivers to articles whose corresponding authors are based in Group A countries and 50% waivers to those who are based in Group B countries as classified by Research4Life (see: https://www.research4life.org/access/eligibility/ ). This section has its own submission website at https://mc.manuscriptcentral.com/e-videos .
